# COHESION: a core outcome set for the treatment of neonatal encephalopathy

**DOI:** 10.1038/s41390-023-02938-y

**Published:** 2023-12-22

**Authors:** Fiona A. Quirke, Shabina Ariff, Malcolm R. Battin, Caitlin Bernard, Linda Biesty, Frank H. Bloomfield, Mandy Daly, Elaine Finucane, Patricia Healy, David M. Haas, Jamie J. Kirkham, Vincent Kibet, Sarah Koskei, Shireen Meher, Eleanor J. Molloy, Maira Niaz, Elaine Ní Bhraonáin, Christabell Omukagah Okaronon, Matthew J. Parkes, Farhana Tabassum, Karen Walker, James W. H. Webbe, Declan Devane

**Affiliations:** 1 Health Research Board Neonatal Encephalopathy PhD Training Network (NEPTuNE), Dublin, Ireland; 2 Health Research Board – Trials Methodology Research Network (HRB-TMRN), University of Galway, Galway, Ireland; 3 School of Medicine, University of Limerick, Limerick, Ireland; 4 Department of Paediatrics & Child Health, Aga Khan University, Karachi, Pakistan; 5 Department of Newborn Services, Auckland District Health Board, Auckland, New Zealand; 6 Department of Obstetrics and Gynecology, Indiana University, Indianapolis, IN US; 7 Evidence Synthesis Ireland, University of Galway, Galway, Ireland; 8 Liggins Institute, University of Auckland, Private Bag 92019, Auckland, 1142 New Zealand; 9 Advocacy and Policymaking, Irish Neonatal Health Alliance, Wicklow, Ireland; 10 Centre for Biostatistics, The University of Manchester, Manchester Academic Health Science Centre, Manchester, UK; 11 Moi University, Cheptiret, Kenya; 12 Birmingham Women’s and Children’s NHS Foundation Trust, Birmingham, UK; 13 Department of Paediatrics and Child Health, Trinity College Dublin, Dublin, Ireland; 14 Department of Neonatology, Children’s Hospital Ireland at Crumlin and Tallaght, Dublin, Ireland; 15 Department of Neonatology, Coombe Women and Infants University Hospital, Dublin, Ireland; 16 Family Support Liaison, Irish Neonatal Health Alliance, Wicklow, Ireland; 17 AMPATH, Eldoret, Kenya; 18 Centre for Statistics in Medicine; Nuffield Dept of Orthopaedics Rheumatology and Musculoskeletal Science, University of Oxford, Oxfordshire, UK; 19 Centre of Excellence in Women and Child Health, Aga Khan University, Karachi, Pakistan; 20 Department of Newborn Care, Royal Prince Alfred Hospital, Sydney, NSW Australia; 21 Faculty of Medicine and Health, University of Sydney, Sydney, NSW Australia; 22 The George Institute for Global Health, Sydney, NSW Australia; 23 Council of International Neonatal Nurses, Sydney, NSW Australia; 24 Academic Neonatal Medicine, Imperial College London, London, UK; 25 Cochrane Ireland, University of Galway, Galway, Ireland

## Abstract

**Background:**

Heterogeneity in outcomes reported in trials of interventions for the treatment of neonatal encephalopathy (NE) makes evaluating the effectiveness of treatments difficult. Developing a core outcome set for NE treatment would enable researchers to measure and report the same outcomes in future trials. This would minimise waste, ensure relevant outcomes are measured and enable evidence synthesis. Therefore, we aimed to develop a core outcome set for treating NE.

**Methods:**

Outcomes identified from a systematic review of the literature and interviews with parents were prioritised by stakeholders (*n* = 99 parents/caregivers, *n* = 101 healthcare providers, and *n* = 22 researchers/ academics) in online Delphi surveys. Agreement on the outcomes was achieved at online consensus meetings attended by *n* = 10 parents, *n* = 18 healthcare providers, and *n* = 13 researchers/ academics.

**Results:**

Seven outcomes were included in the final core outcome set: survival; brain injury on imaging; neurological status at discharge; cerebral palsy; general cognitive ability; quality of life of the child, and adverse events related to treatment.

**Conclusion:**

We developed a core outcome set for the treatment of NE. This will allow future trials to measure and report the same outcomes and ensure results can be compared. Future work should identify how best to measure the COS.

**Impact:**

- We have identified seven outcomes that should be measured and reported in all studies for the treatment of neonatal encephalopathy.
- Previously, a core outcome set for neonatal encephalopathy treatments did not exist.
- This will help to reduce heterogeneity in outcomes reported in clinical trials and other studies, and help researchers identify the best treatments for neonatal encephalopathy.

## Introduction

### Background

Neonatal encephalopathy is a neurological syndrome in term or late preterm infants.^1^ It is characterised by challenges initiating and maintaining respiration, reduced tone and reflexes, seizures, and impaired levels of consciousness.^2^ Neonatal encephalopathy is associated with mortality and long-term disabilities such as cerebral palsy and other developmental impairments.^3–5^

In 2010, Lee et al.^6^ estimated that neonatal encephalopathy associated with intrapartum events affected approximately 1.15 million infants per annum. Different factors can contribute to the development of neonatal encephalopathy, including maternal risk factors, genetic and epigenetic factors, infection, intrapartum events, antepartum events, or sentinel events.^7^ Hypoxia-ischemia is a common cause of neonatal encephalopathy in newborns, contributing to approximately 29% of neonatal encephalopathy.^8^

Treatments for neonatal encephalopathy aim to reduce the risks of adverse long-term outcomes. Therapeutic hypothermia is the standard treatment for hypoxic-ischemic encephalopathy, and involves reducing the infant’s temperature to 33.5 °C for 72 h. A Cochrane systematic review of 11 randomised controlled trials has shown this therapy reduces death and neurodisability in infants with moderate to severe encephalopathy.^9^ Despite 46% of infants having developed adverse events related to cooling, it is now the standard treatment for neonatal encephalopathy in high-income countries (HiCs).^10,11^

Despite its effectiveness in reducing mortality or neurodisability in HiCs, therapeutic hypothermia has its limitations. Therapeutic hypothermia has a narrow therapeutic window and requires initiation of the treatment within the first six hours of injury to optimise its therapeutic benefit. An early diagnosis is, therefore, crucial.^12^ Whilst mortality rates have reduced substantially since clinical trials of therapeutic hypothermia,^13^ the rate of cerebral palsy remains at 19%.^14,15^ In low- to middle-income country (LMiC) settings, therapeutic hypothermia does not appear to reduce death or disability at 18 months, and resulted in an increased incidence of death.^16^

As a result, treatments are now being investigated as stand-alone treatments or adjuvants to therapeutic hypothermia. Therapies include erythropoietin and darbepoetin.^17–22^ Erythropoietin alone or as an adjuvant to therapeutic hypothermia has not improved survival^18,20–23^ and a recent randomised controlled trial in infants with hypoxic-ischemic encephalopathy found that treatment with erythropoietin in addition to therapeutic hypothermia did not reduce the risk of death or neurodevelopmental impairment compared with placebo and was associated with an increased incidence of serious adverse events.^24^ A single-centre randomised controlled trial of melatonin in uncooled infants was reported to improve survival compared with no melatonin.^25^ However, a systematic review of randomised controlled trials of melatonin treatments for neonatal encephalopathy found no significant reduction in mortality when melatonin was combined with therapeutic hypothermia compared to therapeutic hypothermia alone.^26^ Xenon has been investigated in a randomised controlled trial as an adjuvant to therapeutic hypothermia but did not significantly reduce mortality or abnormal magnetic resonance imaging (MRI) results.^27^ Other treatments, including magnesium sulfate,^28^ have also been investigated but have not demonstrated an effect on death or moderate-to-severe neurodevelopmental disability compared with control infants.

A significant obstacle in determining the effectiveness of new treatments for neonatal encephalopathy is the lack of standardisation in the outcomes measured and reported in randomised trials^29^ which impedes comparing, contrasting and synthesising the findings of trials. This contributes to research waste, and potential delays in introducing useful new treatments, as findings cannot be synthesised to inform optimal care for infants with neonatal encephalopathy.^30,31^ For example, in the Cochrane review of randomised trials for therapeutic hypothermia, it was not possible to analyse a number of a priori secondary outcomes as they were not reported.^9^

This heterogeneity in outcome reporting can be minimised by developing a core outcome set (COS). A COS is an agreed standardised set of outcomes that should be measured and reported as a minimum in all studies related to a particular health condition.^31^ Other outcomes may be measured and reported by trialists, but the COS is the minimum number of outcomes that should be measured and reported in all studies. To ensure uptake of the COS, the COS must be relevant to stakeholders (parents/ caregivers of infants diagnosed and treated for neonatal encephalopathy, healthcare providers, and researchers/ academics).

### Objective

The objective was to develop a COS for use in randomised trials and other studies to evaluate the effectiveness of interventions for treating neonatal encephalopathy.

### Scope

The COS has been developed to apply to research studies, including randomised trials, for all interventions for treating neonatal encephalopathy in HiC and LMiC settings.

## Methods

### Protocol entry

This study was developed in-line with guidance published in the Core Outcome Measures in Effectiveness Trials (COMET) Handbook,^31^ and was registered prospectively on the COMET^32^ database (Registration number 1270). The protocol for this study has been published^33^ and its development followed the standards of Core Outcome Set-STAndards for Development: The COS-STAD recommendations.^34^

### Ethics

Research ethics approval was obtained from the University of Galway (Ireland)(Reference number: 19-Apr-14). Additional ethical approval was also obtained in sites participating in the qualitative interviews with parents (Moi University/Moi Teaching and Referral Hospital Institutional Ethics Review Committee (IREC) (Reference: IREC/ 2016/ 243, Approval Number: 0001874), and Aga Khan University Ethics Research Committee (ERC) (2020-5263-14425)).

### COHESION steering group

The COHESION Steering Group was formed in January 2019. It included neonatologists, obstetricians, midwives, a neonatal nurse practitioner, parent (i.e. public and patient involvement (PPI) representatives), experts in COS development and researchers with expertise in neonatal encephalopathy. The collective knowledge of this group informed the planning, design, and development of this COS.

### Study design

**Fig. 1:**
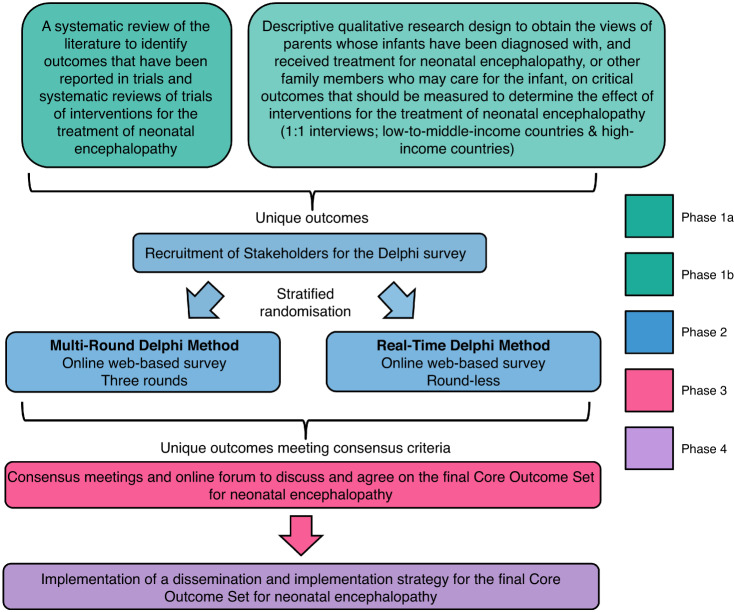
Schematic of COS Development An overview of the phases undertaken to develop the COS.

The development of this COS consisted of five phases (Fig. 1):

- Phase 1a: A systematic review of the literature to identify outcomes that have been reported in trials and systematic reviews of trials of interventions for the treatment of neonatal encephalopathy;^29^
- Phase 1b: A qualitative study using interviews to obtain the views of parents/caregivers whose infants have been diagnosed with, and received treatment for, neonatal encephalopathy on critical outcomes they feel should be measured to determine the effect of treatment(s) for neonatal encephalopathy;^35^
- Phase 2: Development of a preliminary COS (informed by Phases 1 & 2) using the Delphi survey method in which a randomised trial was embedded to evaluate a multi-round Delphi method compared to a Real-Time Delphi method for achieving consensus;
- Phase 3: Consensus meetings to discuss and agree on the final neonatal encephalopathy COS;
- Phase 4: Dissemination of the final COS.

### Participants

We recruited participants for the qualitative study in HiCs and LMiCs through methods described in Quirke et al. 2022.^35^ Participants were parents or other family members who care for, or had cared for, an infant diagnosed with, and received treatment for, neonatal encephalopathy, hypoxic-ischemic encephalopathy, or perinatal/ birth asphyxia.

For the Delphi surveys and the consensus meetings, we recruited:

- Parents of infants diagnosed with and treated for neonatal encephalopathy or hypoxic-ischemic encephalopathy (recruited through parent support networks (see Acknowledgements) and social media)
- Healthcare providers: Neonatal Nurse/Neonatal Nurse Practitioner, Midwives, Obstetricians, Neonatologists, Paediatricians, Neonatal/ Paediatric Neurologists, General Practitioners, Allied Health Professionals (including speech and language therapists, occupational therapists, and physiotherapists with experience of caring for infants with neonatal encephalopathy) and Policymakers recruited through emails Twitter and snowball recruitment from colleagues.
- Researchers/Academics with interest in neonatal encephalopathy research recruited through email, twitter and snowball recruitment from colleagues.

### Information sources

#### Qualitative study

To identify outcomes considered important to measure by parents and caregivers, we conducted interviews. Parents interviewed were from Ireland, the UK, the US, Australia, India, Pakistan, and Kenya. Parents were recruited in HiCs through social media and parent support networks to take part in interviews in English via Zoom teleconferencing software. Parents were recruited in LMiCs with the assistance of designated gatekeepers. Translation processes were developed to recruit parents in Kenya and Pakistan to take part in interviews in their local language in person or by phone. The processes used are described in detail in.^35^

#### Systematic review

We conducted a prospectively registered systematic review to identify outcomes measured and reported in randomised trials and systematic reviews of randomised trials (29). We searched four databases: MEDLINE, Embase, the Cochrane Database of Systematic Reviews (CDSR), Cochrane Central Register of Controlled Trials (CENTRAL), and the World Health Organisation (WHO) International Clinical Trials Registry Platform (ICTRP), between December 2019 and April 2020.

### Consensus process

Participants completed an online Delphi survey. In this part of the COS development process, we embedded a randomised trial to evaluate if different outcomes are prioritised when using a Multi-Round compared to a Real-Time Delphi survey approach in developing a COS for treatments of neonatal encephalopathy.^36^ Recruitment lasted eight weeks to optimise participant numbers. In the Multi-Round Delphi process, including recruitment and all three rounds of the survey lasted 20 weeks. In the Real-Time Delphi process, including recruitment, the survey lasted 14 weeks. Participants were randomly allocated to prioritise outcomes for inclusion in the COS using a Real-Time Delphi (RTD) survey or a three-round Multi-Round Delphi (MRD) survey. This Delphi was run using Calibrum (Surveylet) software. In both surveys, we asked participants to rate outcomes based on their importance for inclusion in the COS, using a 9-point Likert scale, with 1 meaning not important and 9 meaning critical for inclusion in the COS.^31,37^ Participants were also given the option to select ‘I don’t know’. Plain language explanations were provided for each outcome based on previous similar work^38,39^ and in collaboration with parent representatives on the COHESION Steering Group.

Participants were asked to rate 86 outcomes in the surveys. In Round 1 of the MRD and for the first five weeks of the RTD, participants were given the opportunity to suggest other important outcomes that were not included in the Delphi surveys. All additional outcomes suggested were reviewed by the COHESION Steering Group, and if they were deemed to be unique and relevant, they were included in Round 2 of the MRD for rating and in the RTD (Appendix 1). The RTD participants were emailed to remind them that they could revisit the survey and re-rate outcomes they had rated previously and the additional outcomes for the first time. In rounds 2 and 3 of the MRD, the participants were shown graphs displaying the distribution of rating scores for each outcome overall and by each stakeholder group in the previous round.

In the RTD, participants could see the graphs showing how the outcomes were rated overall and by each stakeholder group immediately after they had rated an outcome for the first time. RTD participants could also re-visit the survey and see updates on how the outcomes were rated.

### Consensus definition

Pre-defined consensus criteria for carrying outcomes forward for discussion at the consensus meetings and/ or retaining outcomes in the survey was applied (Table 1). Outcomes that met the criteria for ‘consensus out’ were excluded from the Delphi after week 5 of the RTD and after the close of round 2 in the MRD.

**Table 1:** Consensus criteria for outcomes in the Delphi surveys.

Consensus Classification	Description	Definition
Consensus in (parent-weighted vote)	Consensus that the outcome should be included in the core outcome set	70% or more participants overall scoring as 7 to 9 AND <15% participants scoring a 1 to 3 OR >70% or more of parent group scoring as 7 to 9
Consensus out	Consensus that the outcome should not be included in the COS	50% or fewer participants scoring as 7 to 9 in each stakeholder group
Neither consensus in nor consensus out (consensus undetermined)	Uncertainty about the importance of the outcome, so retain for next round	Anything else

Only participants who completed round 1 of the MRD were invited to complete round 2. Likewise, only those who completed round 2 were invited to complete round 3.

### Consensus meetings

Stakeholders were given the option to express their interest in attending a consensus meeting from the beginning of the RTD and in round 2 of the MRD. A representative group of each stakeholder group (parents/ caregivers, healthcare providers, and researchers/ academics) was sought for the consensus meetings from those who completed all three rounds of the MRD and the RTD. To ensure stakeholders from various countries were present at the consensus meetings, the invitation was extended to healthcare providers and researchers/ academics who did not complete the Delphi but who had expertise and were from low- to middle-income countries.

Consensus on the final outcomes to be included in the COS was achieved through three online meetings held on Zoom in Jan and April 2022. Two initial consensus meetings were held in January to accommodate participants from different time-zones. This was to try to address the difficulties outlined by Gargon et al.^40^ in having international representation at consensus meetings for COS development. The same outcomes were discussed in both meetings. An additional consolidation consensus meeting was held in April to discuss outcomes that were prioritised differently between the two meetings.

Prior to the meeting, participants were sent an information guide containing all outcomes emerging from the Delphi surveys, including consensus scores by stakeholder group, bar charts indicating the distribution of scores by stakeholder group, and plain-language explanations of each outcome. This guide also outlined the agenda of how the meeting would be run, how to use the Zoom platform, and included a scoring sheet whereby participants could reflect on the outcomes and prepare their answers on whether an outcome was ‘not important’, ‘important’ or ‘critical’ for inclusion in the COS, in advance of the meeting.

Outcomes that were rated as either ‘Consensus in’ or ‘Neither consensus in nor consensus out’ from the surveys were carried forward for discussion at the consensus meetings (Appendix 2). Outcomes voted to be excluded from the Delphi survey were also presented to participants who were asked if they agreed with the exclusion of the outcomes. After a detailed discussion on each outcome that was rated either ‘consensus in’ or ‘neither consensus in nor consensus out’, all participants were asked to vote on each outcome as ‘not important’, ‘important’, or ‘critical’ for inclusion in the final COS. The consensus criterion used at the meeting to determine whether an outcome should be included in the final COS was defined, before the meetings, as ≥80% of the consensus meeting participants scoring an outcome as ‘critical’ for inclusion. Anonymous voting was facilitated by participants using the voting function on Zoom.

We had initially planned to populate an online discussion forum to facilitate participants to discuss outcomes prioritised differently at the two consensus meetings and to cast a final vote for inclusion or exclusion in the final COS. Based on the rich discussion in the consensus meetings, we decided to host a consolidation consensus meeting to facilitate discussion of outcomes voted as critical by either of the two previous consensus meetings.

Before the final meeting, all participants were sent a summary document of the outcomes considered critical for inclusion in the COS by both meetings. This summary included discussion points from both meetings on the outcomes.

## Results

The results of this COS development process are reported using the COS-STAR (Core Outcome Set–STAndards for Reporting) guidelines^41^ (Appendix 3).

### Participants

#### Qualitative interviews

We undertook interviews with *n* = 25 parents, from seven countries (Table 2).

**Table 2:** Qualitative interviews participant numbers, countries and country income status.

Number of participants	Country	Income Status of Country
*n* = 11	Ireland	HiC
*n* = 1	Australia	
*n* = 1	USA	
*n* = 1	UK	
*n* = 1	India	LMiC
*n* = 8	Kenya	
*n* = 5	Pakistan	

#### Delphi surveys

In the Delphi process, 222 participants were randomised to take part from 24 different countries (Table 3).

**Table 3:** Delphi survey participant numbers, countries and country income status.

Number of participants	Country	Income Status of Country
*n* = 12	Australia	HiC
*n* = 5	Canada	
*n* = 2	Germany	
*n* = 48	Ireland	
*n* = 1	Italy	
*n* = 2	Netherlands	
*n* = 8	New Zealand	
*n* = 2	Norway	
*n* = 49	UK	
*n* = 1	United Arab Emirates	
*n* = 55	USA	
*n* = 2	Brazil	LMiC
*n* = 3	Chile	
*n* = 1	Colombia	
*n* = 1	Costa Rica	
*n* = 8	Ecuador	
*n* = 8	India	
*n* = 1	Kenya	
*n* = 3	Mexico	
*n* = 1	Pakistan	
*n* = 2	Romania	
*n* = 1	Thailand	
*n* = 2	Turkey	
*n* = 4	Uganda	

#### Consensus meetings

Participants representing different stakeholder groups took part in the consensus meetings from 9 different countries (Table 4).

**Table 4:** Participants who attended the COHESION Consensus Meetings and the countries they represented.

Initial Consensus Meeting 1	Initial Consensus Meeting 2	Consolidation Consensus Meeting
Neonatologist (*n* = 5) Mexico *n* = 2, South Africa *n* = 2, Ireland *n* = 1	Neonatologist (*n* = 2) New Zealand *n* = 1, South Africa *n* = 1	Neonatologist (*n* = 3) New Zealand *n* = 2, Ireland *n* = 1
Midwife (*n* = 1) Ireland *n* = 1	Midwife (*n* = 1) Ireland *n* = 1	Midwife (*n* = 1) Ireland *n* = 1
Neonatal Nurse (*n* = 2) UK *n* = 1, India *n* = 1	Neonatal Nurse (*n* = 1) Australia *n* = 1	Neonatal Nurse (*n* = 0)
Neurologist (*n* = 1) Brazil *n* = 1	Neurologist (*n* = 2) Romania *n* = 1	Neurologist (*n* = 1) Romania *n* = 1
Academic (*n* = ) Ireland *n* = 3, USA *n* = 1	Academic (*n* = 4) South Africa *n* = 1, USA *n* = 1, UK *n* = 1, New Zealand *n* = 1	Academic (*n* = 2) South Africa *n* = 1, USA *n* = 1
Researcher (*n* = 3) UK *n* = 1, South Africa *n* = 2	Researcher (*n* = 2) South Africa *n* = 2	Researcher (*n* = 4)South Africa *n* = 1, Mexico *n* = 1, Ireland *n* = 2
Obstetrician (*n* = 1) UK *n* = 1	Obstetrician (*n* = 2) USA *n* = 1, UK *n* = 1	Obstetrician (*n* = 1) UK *n* = 1
Allied Health Professional (*n* = 0)	Allied Health Professional (*n* = 1) USA *n* = 1	Allied Health Professional (*n* = 1) USA *n* = 1
Parents (*n* = 4) UK *n* = 2, Canada = 1, Ireland *n* = 1,	Parents (*n* = 5) UK *n* = 4, USA *n* = 1	Parents (*n* = 7) UK *n* = 5, Ireland *n* = 1, Canada *n* = 1

### Outcomes

#### Qualitative interviews

Parents identified 54 outcomes as important to measure in the treatment of neonatal encephalopathy.^35^ These outcomes mapped to 16 domains (neurological, respiratory, gastrointestinal, cardiovascular, motor development, cognitive development, development (psychosocial), development (special senses), development (speech and social), other organ outcomes, survival/ living outcomes, patient-reported outcomes, long-term disability, hospitalisation, parent-reported outcomes, adverse events).

#### Systematic review

**Fig. 2:**
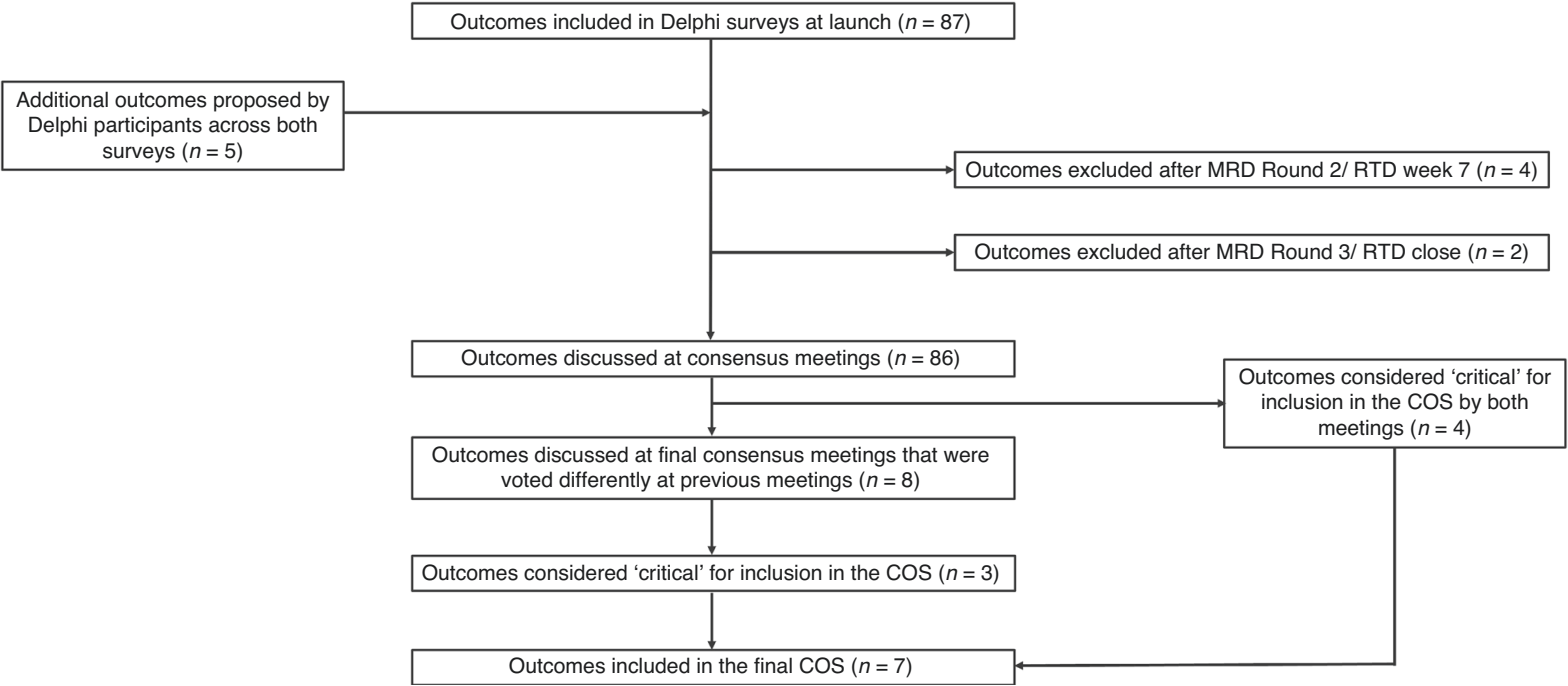
Outcomes identified and prioritised in the COHESION Study.

The systematic review is described in detail elsewhere.^29^ The systematic review identified 66 unique outcomes (Fig. 2, also see Appendix 4), which were grouped into 18 domains (neurological, cardiovascular, respiratory, gastrointestinal, motor development, cognitive development, developmental (special senses), developmental (speech & social), development (psychological), long-term disability outcomes, infection outcomes, hospitalisation, other organ outcomes, patient-reported outcomes, survival/ living outcomes, healthcare utilisation, adverse events, and parent-important outcomes).^29^ There were 33 outcomes identified in the systematic review that were not identified by parents in our qualitative interviews. A further 33 outcomes were common to the systematic review and qualitative interviews. There were also 21 outcomes unique to the interviews that were not captured in the systematic review.

#### Delphi surveys

Overall, *n* = 269 participants accessed the online survey portal. Of these, *n* = 222 completed the initial survey which involved providing consent, demographic information and selecting a ‘role’ to allocate their stakeholder group. Participants were then randomised to either the MRD or the RTD (1:1 ratio) based on their stakeholder group (e.g. parents/ caregivers, healthcare providers, or researchers/ academics). The protocol for this randomised trial is available (36), with results expected to be published shortly. The breakdown of stakeholders who completed each survey are indicated in Table 3. The retention rate overall for the Real-Time Delphi (i.e., the number of participants who completed the survey over those who started as a percentage) was 83%. For the Multi-Round Delphi, the retention rate for all participants completing the three rounds of the survey was 54%. The list of outcomes at the beginning of the Delphi surveys and subsequent suggested outcomes are found in the supplementary information. After the 66 outcomes from the systematic review and the 54 outcomes from the qualitative interviews were combined and duplicates removed, 87 outcomes remained for inclusion in the Delphi surveys (Appendix 4).

At the close of both the MRD and the RTD, the consensus criterion for ‘consensus in’ was met for 48 outcomes, and the consensus criterion ‘Neither consensus in nor consensus out (undetermined consensus)’ was met for 38 outcomes (Appendix 4).

#### Consensus meetings

Two consensus meetings with different participants were held in January 2022. Eight outcomes were prioritised for inclusion in the final core outcome set from each meeting. Four of the eight outcomes prioritised by participants in each meeting were the same and four were unique to each meeting, i.e. 12 outcomes in total. As there were differences of opinion between the stakeholders in each meeting, 12 outcomes were brought forward to the consolidation consensus meeting at which, using the same voting criteria as per each of the two prior consensus meetings, seven outcomes were judged critical for inclusion in the final COS for the treatment of neonatal encephalopathy. Five outcomes discussed at this meeting were not included in the final COS. Electroencephalogram (EEG) abnormalities were discussed extensively as an important and valuable method of determining brain activity following treatment. However, due to a lack of availability in low-resource settings, it was deemed unreasonable to expect this outcome to be measured in studies conducted in LMiCs. Similarly, the outcome of ‘neonatal seizures’ was discussed extensively and was important to both parents and healthcare providers. However, at the consolidation consensus meeting, as seizures are not present in all infants with neonatal encephalopathy, this outcome did not reach the consensus threshold for inclusion in the final COS. The outcome ‘severity of encephalopathy’ was also considered important to measure; however, it was not voted as critical to include in the final core outcome set. Instead, an outcome of ‘neurological status at discharge’ was proposed to encompass this outcome and to establish a time-point to measure this outcome. The outcome ‘general gross motor ability’ was extensively discussed; however, it was judged that significant motor deficits would be overlapping with the outcome of ‘cerebral palsy’ and that this outcome was more critical for inclusion in the final COS. Feeding was also discussed and acknowledged as an important indicator of neurological function in infants. This outcome was suggested to be included in the ‘how’ to measure ‘neurological function at discharge’, which is included in the final COS. Although the ‘quality of life of the parents’ was acknowledged as extremely important in the care of infants diagnosed with neonatal encephalopathy, it was decided that this outcome was more ‘important’ than ‘critical’ for measuring and reporting in all randomised trials for the treatment of neonatal encephalopathy. In addition, participants felt that that the quality of life of the child was considered more critical to include in the COS.

### Core outcome set

The final core outcome set comprises the following outcomes:

- Survival
- Brain injury on imaging
- Neurological status at discharge
- Cerebral palsy
- General cognitive ability
- Quality of life of the child
- Adverse events

Many of the outcomes that were judged not critical for inclusion in the COS were judged important, just not critical for inclusion in the COS (either at the initial or final meeting). These outcomes are available in the Supplementary Information so that researchers can choose whether to include these in addition to the 7 outcomes included in the COS.

## Discussion

In keeping with the methodological guidance for the development of COS,^31^ this COS should be used in future studies evaluating neonatal encephalopathy treatments. This will help to reduce research waste because the findings of different treatments can be compared, and contrasted, and the most effective treatments better determined.

It is important to note that the COS represents the minimum set of outcomes that should be measured and reported in all treatment trials. Other outcomes (Appendix 5) can be measured and reported if deemed relevant, but they should be in addition to the 7 core outcomes.

This study followed the study protocol with three notable exceptions.^33^ In our systematic review, we also included studies for treating perinatal/ birth asphyxia in addition to neonatal encephalopathy or hypoxic-ischemic encephalopathy. We did not extract data on the timing of measurement of the outcomes in the systematic review. We decided to host a consolidation consensus meeting to facilitate discussion in the process of achieving consensus on a final COS for the treatment of neonatal encephalopathy.

This study involved international collaboration of parents/ caregivers of infants diagnosed and treated for neonatal encephalopathy, healthcare providers, and researchers/ academics with interest in neonatal encephalopathy. Input from key stakeholders and experts in the field of neonatal encephalopathy research increases the likelihood that this COS will be measured and reported in future trials for treatments. Ultimately, this will help inform clinical practice and care for infants with neonatal encephalopathy.

Identifying outcomes from our systematic review ensured the outcomes considered important to healthcare providers and researchers were included in the Delphi consensus surveys. Interviewing parents from various HiCs and LMiCs helped us identify outcomes that were not measured and reported in trials for treatments. This ensured that outcomes scored and discussed were representative of the relevant stakeholders’ opinions and expertise.

Although we conducted a randomised trial of two Delphi survey types (MRD vs. RTD), outcomes were retained if they were considered critical for inclusion by one or both survey arms. In this way, the trial itself did not impact on outcomes being retained or removed throughout the survey process. Investigating alternative methods to the multi-round Delphi approach may help inform decisions on optimal approaches.

We recognise that some outcomes may be difficult to measure, for example quality of life and cerebral palsy. Nonetheless, the results of long-term outcomes are needed in trials to understand the effectiveness of treatments. If an outcome cannot be measured, for example due to loss to follow-up of trial participants or low resource availability. It is important to note that there was strong consensus among stakeholders, including parents, that these outcomes should be measured or stated why it was not possible to measure this outcome this should be reported. Subsequent work is needed to identify *how* to measure the core outcomes prioritised in this study.^42^

This COS compliments the COS for neonatology work by Webbe et al.^38^ Five out of the seven outcomes included in our COS appear in the COS for neonatology. The unique outcomes in this COS are neurological status at discharge and cerebral palsy. This suggests that for broader COS, like that for neonatology, which encompasses several conditions such as neonatal encephalopathy, preterm conditions, neonatal sepsis etc., it may be possible to develop more specific COS for particular conditions within the general sphere covered by the broad COS by conducting a modified COS development process such as a Delphi survey evaluating the current COS or consensus meeting with key stakeholders alone.

### Strengths and limitations

In identifying the outcomes measured and reported in randomised trials of treatments for neonatal encephalopathy, we limited our search to published clinical trials and systematic reviews. We included only English-language papers in our systematic review, due to a lack of resources to enable the translation of papers. However, given the large number of papers reviewed (116 papers), and the large international panel of stakeholders who took part in the qualitative interviews (*n* = 25) and Delphi survey (*n* = 222), only five additional outcomes were suggested by participants of the Delphi survey that were not already included. This gave us confidence that we had identified the outcomes considered important to parents, healthcare providers and researchers/academics from our interviews and systematic search of published trials. We acknowledge these pragmatic decisions as potential limitations.

A further limitation of our work is conducting the Delphi surveys and consensus meetings in English only. Despite the contribution of stakeholders from LMiCs in all stages of this COS development, limiting some stages to English speakers may impact outcomes included in the final COS. This has been explored in other COS;^43^ however, budget and resource limitations are likely to be a barrier to adopting this approach in many COS. However, by including non-English speakers from LMiCs in the identification of important outcomes in the beginning of the COS development process, the risk of not capturing the views of those in LMiCs was minimised.

Recruiting a diverse group of stakeholders ensures that the outcomes included in the final COS are relevant to all those involved and impacted by the treatment of neonatal encephalopathy (i.e. parents/ caregivers, healthcare providers, and researchers/ academics). Although the number of researchers/ academics taking part in the Delphi surveys was lower than the parents/ caregivers and healthcare providers, many of the healthcare providers also had experience in conducting research. In limiting participants to select one primary stakeholder identity, the input of researchers is likely to be higher than documented. The large number of parent/ caregivers taking part in the Delphi survey and consensus meetings ensures the COS is representative of their perspective and ensures meaningful patient inclusion in this overall COS development.

It is recognised that for COS, the involvement of stakeholders from various countries contributes to the validity of the COS and makes it easier to implement the COS into studies in low-resource settings as well as high-resource settings.^44^ For this reason, we tried to ensure that LMiC participants were included in all stages of this COS development. Recruitment of parents/ caregivers from LMiCs to take part in qualitative interviews was supported by a process of translating all necessary documentation and interview guide implemented. By collaborating with experienced qualitative researchers in LMiC sites, the process of recruitment, translation, conducting interviews, translating and transcribing results was established successfully. Providing alternative dates and times for consensus meetings ensured stakeholders from countries in different time zones could participate.

The rich discussion facilitated by deciding to host the consolidation consensus meeting is a key strength of this study overall. Stakeholders were given an additional opportunity to discuss why outcomes were important to them and to decide what outcomes were critical for inclusion rather than important. This increased our confidence that the outcomes included in the final COS are important and relevant to stakeholders.

We have chosen the term Neonatal Encephalopathy (NE) as the descriptor for the condition under investigation. This decision is informed by a multifaceted understanding of the condition’s nature and in contemporary discourse in both clinical and research communities. While our choice is consistent with recommendations from several authoritative bodies, including the American College of Obstetricians and Gynecologists Task Force on Neonatal Encephalopathy,^45^ it also aligns with the broader understanding that NE represents a syndrome of disturbed neurological function in term or late preterm neonates in the first few days of life.

While some view Hypoxic Ischemic Encephalopathy (HIE) as a distinct subset of NE, specifically caused by inadequate blood flow and oxygen to the brain, many prominent bodies, including the American Academy of Pediatrics and the British Association of Perinatal Medicine (BAPM), advocate for using NE until the exact aetiology is determined. This stance, echoed by Hurley et al.,^46^ emphasises the importance not assuming a specific cause until it is definitively established, especially given the considerable variation in the use of the terms NE and HIE across clinical studies.

It is crucial to note that our study did not determine if every participant and contributor to the COS strictly adhered to the HIE criteria. Consequently, we cannot conclusively state that this COS pertains exclusively to HIE. We acknowledge that this choice of terminology may not align with all perspectives in the field, and we respect the ongoing discussions and debates surrounding the use of NE versus HIE. However, editorials in Pediatric Research underscore the importance of using NE as a more encompassing term that includes various causes of encephalopathy.^47,48^ These discussions highlight that the terms NE and HIE are frequently used interchangeably, promoting the use of NE as a broader term that doesn’t specify a particular aetiology. This approach allows clinicians to explore the cause more definitively, avoiding potential pitfalls, such as attributing a cause (hypoxia-ischemia) to the disorder (encephalopathy) without concrete evidence.

Findings from a recent systematic review by Hurley et al. further bolster our choice. In their review, Hurley et al. analysed 67 randomised controlled trials (RCTs) spanning 20 countries, involving 6412 participants, and published from 1998 to 2022. Their findings revealed a notable variation in terminology across studies: HIE was the predominant term in 56 out of 67 studies, while NE was used in only 16. Despite this, the most commonly employed inclusion criteria across these studies were Apgar scores, metabolic acidosis, reduced level of consciousness, reduced tone, and abnormal reflexes This underscores the need for a unified term like NE, which can encompass the diverse clinical manifestations and aetiologies observed across these studies.

Additionally, a recent editorial by Molloy et al.^49^ offers a comprehensive perspective on the nuances of NE and its distinction from HIE. It too emphasises that NE is a clinical syndrome observed in the first week after birth in infants born at or beyond 35 weeks of gestation, characterised by disturbed neurological function. This broad clinical definition does not specify subgroups or aetiology, which has led to ambiguities in the field. It acknowledges that term NE and HIE are often used interchangeably, even though HIE is essentially a subgroup of NE. This overlap in terminology has, the authors claim, posed challenges in case definition, collaborative research, and data synthesis, and lead to confusion among families and caregivers alike. The editorial underscores the need for consensus on diagnosis, terminology, and classification to ensure clarity and precision in understanding and managing these conditions. The insights from this editorial further bolster our argument for using the term NE, emphasising its broader scope and the complexities associated with its diagnosis and classification. Similar recent insights into neuroprotective therapies shed light on the intricate nature of NE and the challenges surrounding its terminology.^50^ The editorial underscores that while therapeutic hypothermia has revolutionised the treatment of NE, the condition remains multifaceted, with a spectrum of underlying causes. Given that a significant proportion of infants with NE still face adverse outcomes despite current treatments, it is clear that NE is not a singular condition. This complexity reinforces our decision to use the term NE as a more inclusive descriptor that refrains from assigning a premature aetiology.

In our commitment to transparency and constructive dialogue, we recognise the ongoing debates and the multifaceted nature of this issue. Our decision to adopt the term NE aligns with our objective to encompass a wide spectrum of encephalopathy causes and is bolstered by current discussions and in-depth insights in the field. We are confident that our approach will enrich the ongoing discourse, paving the way for clarity and consensus that will be advantageous for both research and clinical practice. Typically, COS are developed for use in randomised trials for a condition. However, this standardised set of outcomes may also help in routine care to ensure that the measured outcomes are important to all those involved in caring for infants with this condition.

We will disseminate this COS by sharing the final COS with all participants, relevant publications, presentations at relevant conferences, website updates, newsletters, press releases and social media updates.

## Conclusion

The COHESION Study has identified a COS for use in randomised trials and other studies to evaluate the effectiveness of interventions for treating neonatal encephalopathy. Using this COS in future studies will ensure that the outcomes measured and reported are relevant to all those involved in treating and caring for infants diagnosed with neonatal encephalopathy. Standardising the outcomes measured and reported in studies will facilitate better synthesis of results to help researchers and healthcare providers decide the optimal treatment for infants with neonatal encephalopathy.

### Supplementary Information

Appendix

## Data Availability

The datasets generated during and/or analysed during the current study are available from the corresponding author on reasonable request.
