# Supplementary material for: COHESION: a core outcome set for the treatment of neonatal encephalopathy
Source: Pediatr Res. 2023 Dec 22;95(4):922–30. doi: 10.1038/s41390-023-02938-y (PMC10920183; doi:10.1038/s41390-023-02938-y)
Supplement: Supplementary file 1 — Appendix [file 41390_2023_2938_MOESM1_ESM.pdf]

Appendix 1: List of outcomes included in the Delphi survey (note (*) denotes that the outcome was suggested in the Delphi process, (^) denotes that the outcome was excluded in the Delphi process.

- Brain injury on imaging
- Electroencephalogram (EEG) abnormalities
- Biomarker evidence of brain injury
- Intracranial haemorrhage
- Severity of encephalopathy
- Absence of neonatal reflexes
- Gag reflex (absent)
- Swallow (absent)
- Neonatal seizures
- Sleep disorders
- Normal tone
- Abnormal changes in heart rate or rhythm
- Coagulopathy
- Thrombosis
- Hypertension (and management)
- Hypotension (and management)
- Haematological variables
- Myocardial dysfunction
- Cardiac ischemia
- Need for neonatal resuscitation
- Oxygen requirement
- Need for mechanical ventilation
- Need for inhaled nitric oxide
- Need for extracorporeal membrane oxygenation use
- Respiratory distress
- Ability to breathe normally and unaided
- Persistent pulmonary hypertension
- Lung air leaks^
- Pulmonary haemorrhage
- Feeding intolerance
- Need for tube feeding
- Necrotising enterocolitis
- Oral feeding ability
- Need for surgical treatment of gastro-oesophageal reflux disease^
- Meconium Passage^
- General gross motor ability
- Fine motor development
- Ability to undertake sport^
- Need for physiotherapy*
- General cognitive ability
- Normal memory
- Child mental health

- Heightened sensory sensitivity
- Hearing impairment
- Visual impairment
- Speech delay
- Ability to make noises/ verbalise
- General communication ability
- Psychological development
- Self-esteem
- Behavioural challenges
- Development of Attention Deficit/ Hyperactive Disorder (ADHD)*
- Development of Autism Spectrum Disorder (ASD)*
- Cerebral palsy
- Epilepsy
- Requirement for antiepileptic drugs at discharge
- Need for occupational therapy
- Survival
- Suffering
- Growth
- Full recovery from acute illness
- Requirement for analgesics
- Quality of life of patient
- Sepsis
- Pyrexia
- Duration of neonatal stay
- Readmission in childhood
- Healthcare costs
- Need for multiple operations
- Adverse event related to treatment
- Poor renal function
- Hepatic dysfunction
- Electrolyte disturbance
- Metabolic acidosis
- Pneumonia
- Hyperglycemia^
- Hypoglycemia
- Hypoxia
- Jaundice*^
- Multi-organ dysfunction
- Emotional impact on parents
- Parental involvement in care
- Parental attachment with their baby
- Parental senses of loss of normal
- Uncertainty for future wellbeing
- Parental psychological impact of NICU experience
- Impact of child’s condition on parent’s relationship
- Financial burden of healthcare costs of care for an infant on parents
- Parental ability to work

- Impact of child’s condition and neonatal intensive care unit (NICU) experience on wider family (stress, disappointment, sadness, grief etc.)
- Effective communication
- Impact on family decision to have other children*

Appendix 2: Outcomes brought forward to the consensus meetings.

Outcome	*Consensus result*
**Neurological**	
Brain injury on imaging	in
EEG abnormalities	undetermined
Biomarker evidence of brain injury	undetermined
Severity of encephalopathy	in
Absence of neonatal reflexes	in
Gag reflex (absence)	in
Swallow (absent)	in
Neonatal seizures	in
Sleep Disorders	undetermined
Normal Tone	in
Intracranial haemorrhage	in
**cardiovascular**	
Abnormal changes in heart rate or rhythm	undetermined
Coagulopathy	undetermined
Thrombosis	undetermined
Hypotension	undetermined
Hypertension	undetermined
Haematological variables	undetermined
Myocardial Dysfunction	undetermined
Cardiac Ischemia	undetermined
**adverse events**	
Adverse events	in
**respiratory**	
Need for neonatal resuscitation	in
Oxygen requirement	in
Need for mechanical ventilation	in
Need for extracorporeal membrane oxygenation	undetermined
Need for inhaled nitric oxide	undetermined
Respiratory distress	in
Ability to breathe normally and unaided	in
Persistent pulmonary hypertension	undetermined
Pulmonary haemorrhage	undetermined

**gastrointestinal**	
Feeding intolerance	undetermined
Need for tube feeding	in
Necrotising enterocolitis	undetermined
Oral feeding ability	in
**motor development**	
General gross motor ability	in
General fine motor ability	in
Need for physiotherapy	in
**patient-reported outcomes**	
Quality of life of patient	in
**cognitive**	
General cognitive ability	in
Normal memory	in
Child mental Health	in
ADHD	undetermined
ADD	in
**development (special senses)**	
Heightened sensory sensitivity	undetermined
Hearing impairment	undetermined
Visual impairment	in
**development (speech & social)**	
Speech delay	in
Ability to make noises/ verbalise	in
General communication ability	in
**development (psychosocial)**	
Psychological development	in
Self esteem	in
Behaviour challenges	in
**infection outcomes**	
Sepsis	undetermined
Pyrexia	undetermined
**long-term disability**	
Cerebral palsy	in
Epilepsy	in
Requirement for antiepileptic drugs at discharge	undetermined
Need for occupational therapy	undetermined
**survival/ living outcomes**	
Survival	in
Suffering	in
Growth	undetermined
Full recovery from acute illness	in
Requirement for analgesics	undetermined
**other organ outcomes**	
Poor renal function	undetermined

Hepatic dysfunction	undetermined
Metabolic acidosis	undetermined
Pneumonia	undetermined
Electrolyte disturbance	undetermined
Hypoglycaemia	undetermined
Hypoxia	in
Multi-organ dysfunction	in
**parent-important outcomes**	
Emotional impact on parents	in
Parental involvement in care	in
Parental attachment with their baby	in
Parental sense of loss of normal	in
Uncertainty for future wellbeing	in
Parental psychological impact of NICU experience	in
Impact of child's condition on parent's relationship	in
Financial burden of healthcare costs of care for an infant parent	in
Parental ability to work	undetermined
Impact of child's condition on NICU experience	undetermined
Effective communication	in
Impact on family decision to have other children	undetermined
**hospitalisation outcomes**	
Duration of neonatal stay	undetermined
Readmission in childhood	in
**healthcare utilisation**	
Healthcare costs	undetermined
Need for multiple operations	undetermined

Appendix 3: COS-STAR Checklist.

SECTION/TOPIC	ITEM No.	CHECKLIST ITEM	PAGE NUMBER
TITLE/ABSTRACT			
Title	1a	Identify in the title that the paper reports the development of a COS	1
Abstract	1b	Provide a structured summary	4
INTRODUCTION			
Background and Objectives	2a	Describe the background and explain the rationale for developing the COS.	5 - 7

	2b	Describe the specific objectives with reference to developing a COS.	7
Scope	3a	Describe the health condition(s) and population(s) covered by the COS.	7
	3b	Describe the intervention(s) covered by the COS.	7
	3c	Describe the setting(s) in which the COS is to be applied.	7
METHODS			
Protocol/Registry Entry	4	Indicate where the COS development protocol can be accessed, if available, and/or the study registration details.	7 & 8
Participants	5	Describe the rationale for stakeholder groups involved in the COS development process, eligibility criteria for participants from each group, and a description of how the individuals involved were identified.	9 & 10
Information Sources	6a	Describe the information sources used to identify an initial list of outcomes.	10 & 11
	6b	Describe how outcomes were dropped/combined, with reasons (if applicable).	Figure 2
Consensus Process	7	Describe how the consensus process was undertaken.	11 & 12
Outcome Scoring	8	Describe how outcomes were scored and how scores were summarised.	12 - 14
Consensus Definition	9a	Describe the consensus definition.	12
	9b	Describe the procedure for determining how outcomes were included or excluded from consideration during the consensus process.	Table 1
Ethics and Consent	10	Provide a statement regarding the ethics and consent issues for the study.	8
RESULTS			
Protocol Deviations	11	Describe any changes from the protocol (if applicable), with reasons, and describe what impact these changes have on the results.	16 & 17, 18 - 20
Participants	12	Present data on the number and relevant characteristics of the people involved at all stages of COS development.	14 & 15
Outcomes	13a	List all outcomes considered at the start of the consensus process.	15 - 18

	13b	Describe any new outcomes introduced and any outcomes dropped, with reasons, during the consensus process.	Figure 2
COS	14	List the outcomes in the final COS.	18
DISCUSSION			
Limitations	15	Discuss any limitations in the COS development process.	20 - 22
Conclusions	16	Provide an interpretation of the final COS in the context of other evidence, and implications for future research.	18 - 23
OTHER INFORMATION			
Funding	17	Describe sources of funding/role of funders.	31
Conflicts of Interest	18	Describe any conflicts of interest within the study team and how these were managed.	31

Appendix 4: Schematic of outcomes identified and prioritised in the COHESION Core Outcome Set development process.

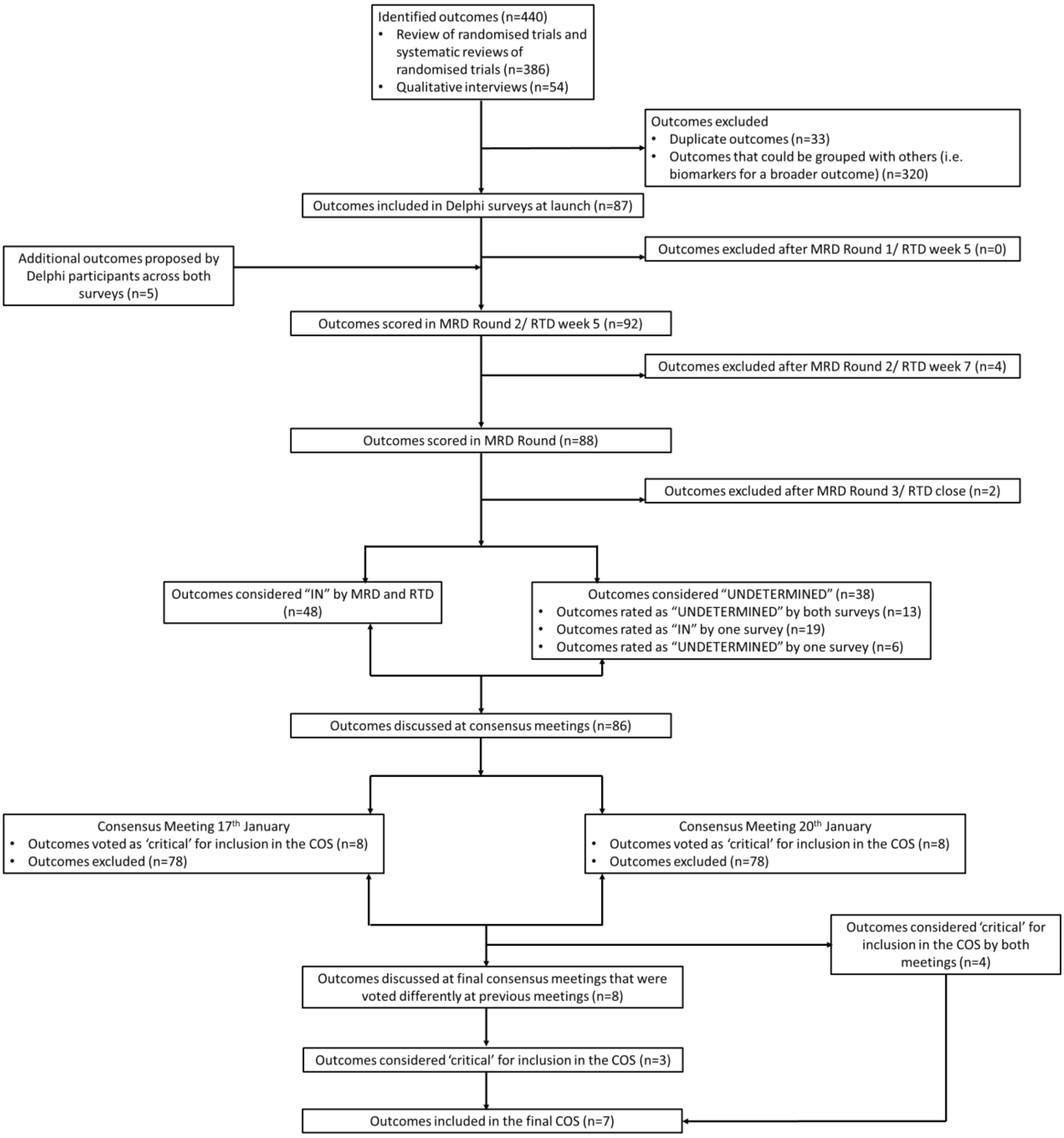

Appendix 5: Outcomes considered important to measure in future trials but not critical for inclusion in the COS.

- Electroencephalogram (EEG) abnormalities
- Neonatal seizures
- Biomarker evidence of brain injury
- Absence of neonatal reflexes
- Gag reflex (absent)
- Swallow (absent)
- Severity of encephalopathy
- Normal tone
- Abnormal changes in heart rate or rhythm

- Coagulopathy
- Myocardial dysfunction
- Cardiac ischemia
- Respiratory distress
- Need for mechanical ventilation
- Oxygen requirement
- Need for extracorporeal membrane oxygenation (ECMO)
- Need for inhaled nitric oxide (NO)
- Persistent pulmonary hypertension
- Ability to breathe normally and unaided
- Pulmonary haemorrhage
- Suffering
- Growth
- Sepsis
- Pyrexia
- Full recovery from acute illness
- Healthcare costs
- Multi-organ dysfunction
- Duration of neonatal stay
- Readmission in childhood
- Poor renal function
- Hepatic dysfunction
- Metabolic acidosis
- Pneumonia
- Hypoglycemia
- Hypoxia
- Parental quality of life
- Feeding ability
- General fine motor ability
- Need for physiotherapy
- Normal memory
- Child mental health
- Attention deficit hyperactivity disorder (ADHD)
- Heightened sensory sensitivity
- Hearing impairment
- Visual Impairment
- Speech delay
- Ability to make noises/ verbalise
- General communication ability
- Psychological development
- Self esteem
- Behavioural challenges
- Epilepsy
- Need for occupational therapy
